# Transethnic Meta-Analysis of Genomewide Association Studies

**DOI:** 10.1002/gepi.20630

**Published:** 2011-11-28

**Authors:** Andrew P Morris

**Affiliations:** Wellcome Trust Centre for Human Genetics, University of OxfordOxford, United Kingdom

**Keywords:** meta-analysis, transethnic, genomewide association study, diverse populations, Bayesian partition model, fine-mapping

## Abstract

The detection of loci contributing effects to complex human traits, and their subsequent fine-mapping for the location of causal variants, remains a considerable challenge for the genetics research community. Meta-analyses of genomewide association studies, primarily ascertained from European-descent populations, have made considerable advances in our understanding of complex trait genetics, although much of their heritability is still unexplained. With the increasing availability of genomewide association data from diverse populations, transethnic meta-analysis may offer an exciting opportunity to increase the power to detect novel complex trait loci and to improve the resolution of fine-mapping of causal variants by leveraging differences in local linkage disequilibrium structure between ethnic groups. However, we might also expect there to be substantial genetic heterogeneity between diverse populations, both in terms of the spectrum of causal variants and their allelic effects, which cannot easily be accommodated through traditional approaches to meta-analysis. In order to address this challenge, I propose novel transethnic meta-analysis methodology that takes account of the expected similarity in allelic effects between the most closely related populations, while allowing for heterogeneity between more diverse ethnic groups. This approach yields substantial improvements in performance, compared to fixed-effects meta-analysis, both in terms of power to detect association, and localization of the causal variant, over a range of models of heterogeneity between ethnic groups. Furthermore, when the similarity in allelic effects between populations is well captured by their relatedness, this approach has increased power and mapping resolution over random-effects meta-analysis. *Genet. Epidemiol*. 2011. © 2011 Wiley Periodicals, Inc.35: 809–;822, 2011.

## INTRODUCTION

Genomewide association studies (GWAS) have been extremely successful in identifying loci contributing genetic effects to a wide range of complex human traits. However, despite this success, the joint effects of these loci typically explain only a small proportion of the heritability [Manolio et al., 2009; McCarthy et al., 2008]. Furthermore, the loci identified through GWAS often extend over hundreds of kilobases, contain many genes and large numbers of variants with indistinguishable signals of association, occurring as a result of linkage disequilibrium (LD) across the region. The challenge is now to identify novel loci that contribute to the “missing” heritability of complex traits, and to refine the location of causal variants within already established loci in order to prioritize genes for followup through functional studies.

The vast majority of GWAS have been undertaken in populations of European descent [Rosenberg et al., 2010]. The availability of European-descent population cohorts, such as those made available by the Wellcome Trust Case Control Consortium [The Wellcome Trust Case Control Consortium, 2007], has expedited the use of “shared controls” between GWAS, reducing the burden of sample collection and genotyping [Zhuang et al., 2010]. Meta-analyses of European-descent GWAS have proved to be profitable in identifying additional complex trait loci by increasing sample size without the cost of additional genotyping [Barrett et al., 2009; Dupuis et al., 2010; Lango Allen et al., 2010; Voight et al., 2010]. This process has been greatly aided by the development of imputation techniques that allow the prediction of genotypes not typed on GWAS chips, but present on a higher density reference panel of phased haplotypes from the same, or a closely related population [Marchini and Howie, 2010]. Appropriate reference panels for European-descent populations have been made available through the International HapMap Project [The International HapMap Consortium, 2007, 2010] and at higher density through the 1000 Genomes Project [The 1000 Genomes Project Consortium, 2010]. These reference panels provide more complete coverage of common genetic variation throughout the genome, and thus will be more likely to explicitly include causal variants than will GWAS genotyping products. However, LD between common variants among European-descent populations will likely continue to hamper fine-mapping efforts, even with the large sample sizes accrued through GWAS meta-analysis.

Two of the key challenges in performing GWAS in other ethnic groups have been the lack of appropriate genotyping products and availability of well-matched imputation reference panels [Jallow et al., 2009]. Initial GWAS chips were designed to preferentially capture common genetic variation in Europeans [Rosenberg et al., 2010]. Underlying differences in the structure of LD between diverse populations reduced the efficiency of these genotyping products in other ethnic groups. However, more recent chips are less biased to European-descent populations, and GWAS are now increasingly undertaken, with great success, in other ethnic groups including Japanese [Kamatani et al., 2010; Kochi et al., 2010; Takata et al., 2010; Uno et al., 2010; Yamauchi et al., 2010], Chinese [Abnet et al., 2010; Chen et al., 2011; Wang et al., 2010], Koreans [Jee et al., 2010], Indian Asians [Chambers et al., 2010] and Africans [Petrovski et al., 2010; Thye et al., 2010]. Furthermore, the 1000 Genomes Project will provide comprehensive reference panels of common variants, and hence permit accurate imputation, in diverse ethnic groups from African, Asian and American, as well as European-descent populations [The 1000 Genomes Project Consortium, 2010].

With the increasing availability of GWAS data from diverse populations, transethnic meta-analysis may offer an exciting opportunity to increase the power to detect novel loci, through increased sample size, as well as to improve the resolution of fine-mapping of causal variants [Cooper et al., 2008; Zaitlen et al., 2010]. The underlying differences in the structure of LD between ethnic groups can be leveraged to amplify the signal of association at the causal variant. In particular, we would not expect that any set of indistinguishable associated variants will be the same in all populations from different ethnic groups. However, the allele frequency spectrum is also highly variable between diverse populations, with the result that a causal variant may be specific, or more relevant, to one ethnic group. For example, the risk allele for a causal variant for cardiomyopathy in *MYBPC3* has 4% frequency in populations from the Indian subcontinent, but is much rarer or not observed in other ethnic groups [Dhandapany et al., 2009]. Furthermore, causal variants may interact with environmental risk factors that differ in exposure between ethnic groups, generating variability in the marginal allelic effect between populations. It is thus not clear that the findings of GWAS will translate from one ethnic group to another, and hence that we might expect considerable heterogeneity in allelic effects between distantly related populations.

Irrespective of the source of genetic heterogeneity, traditional methodology for the meta-analysis of GWAS, as implemented in the GWAMA software [Magi and Morris, 2010], cannot appropriately take account of the resulting variability in allelic effects between ethnic groups. Fixed-effects meta-analysis assumes the allelic effect to be the same in all populations. Conversely, random effects meta-analysis assumes that each population has a different underlying allelic effect. This is also unsatisfactory since we expect populations from the same ethnic group to be more homogeneous than those that are more distantly related. In order to address this challenge, I have developed novel transethnic meta-analysis methodology that takes account of the expected similarity in allelic effects between the most closely related populations by means of a *Bayesian partition model* [Denison and Holmes, 2001; Knorr-Held and Rasser, 2000]. Briefly, for each variant, allelic effects and the corresponding standard errors are estimated within each population under the assumption of an additive model for the reference allele. Populations are then clustered according to their similarity in terms of relatedness (i.e. shared ancestry) and allelic effects at the variant. Populations within the same cluster are assumed to have the same underlying allelic effect. However, clusters are assumed to have different underlying allelic effects, thus allowing for heterogeneity. The methodology has been implemented in the MANTRA (Meta-ANalysis of Transethnic Association studies) software.

In this article, I apply MANTRA to association studies of type 2 diabetes (T2D) from five diverse ethnic groups [Waters et al., 2010], and highlight the evidence of heterogeneity in allelic effects between populations at the *CDKAL1* locus. I demonstrate, by means of simulation, substantial improvements in the performance of MANTRA, compared to traditional fixed-effects meta-analysis, both in terms of power to detect association, and localization of the causal variant, over a range of models of heterogeneity between ethnic groups. Furthermore, I also demonstrate increased power and mapping resolution for MANTRA over random-effects meta-analysis when the pattern of allelic effects between populations is well captured by the Bayesian partition model. These results highlight the potential of MANTRA to detect and fine-map novel loci for complex traits through application to transethnic GWAS.

## METHODS

Consider the results of a series of *N* transethnic GWAS of a continuous or dichotomous trait, ascertained from populations *P*_1_, *P*_2_,…, *P*_*N*_, at a given variant. We denote by *b*_*i*_ and *s*_*i*_ the estimated allelic effect (under an additive model, i.e. log-odds ratio in the context of a dichotomous trait) and corresponding standard error, respectively, of the *i*th study at the variant. In traditional meta-analysis, we typically assume that *b*_*i*_∼*N*(β_*i*_,*s*_*i*_), where β_*i*_ denotes the *i*th population-specific allelic effect.

Under the null model, *M*_0_, of no association of the variant with the trait in *any* population, β = **0**. In a Bayesian framework, the evidence in favor of the alternative model, *M*_1_, corresponding to β≠**0**, can be assessed by means of the *Bayes*' *factor* [Kass and Raftery, 1995], given by


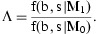


In this expression, *f*(**b**,**s**|*M*) denotes the marginal likelihood of the observed allelic effects under model *M*. This marginal likelihood is given by integration over the unknown model parameters, **θ**, which include the population-specific allelic effects, **β**, and additional hyper-parameters relating to their prior distribution, to be defined later. It thus follows that


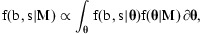
(1)

where the likelihood


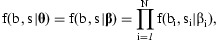


and


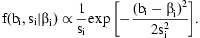
(2)

### BAYESIAN PARTITION MODEL

Under a Bayesian partition model [Denison and Holmes, 2001; Knorr-Held and Rasser, 2000], **β** is determined by the assignment of populations to *ethnic clusters*, referred to as a tessellation, and the corresponding cluster allelic effects, **ψ**. The tessellation is defined by specifying *K* cluster centers, 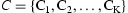, ordered and without replacement from the populations. Remaining populations are then assigned to the “nearest” cluster centre. Here, the distance between the *i*th population, *P*_*i*_, and *k*th cluster centre, *C*_*k*_, is measured by the *F*-statistic (*F*_ST_) or some other metric of allele frequency dissimilarity [Weir and Cockerham, 1984; Weir and Hill, 2002; Wright, 1951]. If a population is equidistant from multiple nearest cluster centers, it is assigned to that with minimum *k*. The tessellation is then given by **T**, where *T*_*ik*_ = 1 if population *P*_*i*_ is assigned to the cluster with centre *C*_*k*_, and 0 otherwise.

For a given tessellation, we can then express the population-specific contribution to the likelihood in Equation (2) as


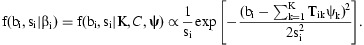
(3)

The special case of a single cluster, *K* = 1, corresponds to no heterogeneity between population-specific allelic effects, and thus can be thought of as a Bayesian implementation of fixed-effects meta-analysis. Furthermore, when *K* = *N*, each population is assigned to a different cluster, and thus can be thought of as a Bayesian implementation of random-effects meta-analysis.

### PRIOR DENSITY FUNCTION

The Bayes' factor, Λ, depends on the prior density function, *f*(**θ**|*M*), of parameters under model *M*. Under the null model, *M*_0_, the population-specific allelic effects are all zero, and hence any clustering of populations is irrelevant. Hence, *f*(**θ**|*M*_0_) = 1 if **β** = **0**, and 0 otherwise. Conversely, under *M*_1_, population-specific allelic effects are determined by the Bayesian partition model. Under this model, the prior density of the number of clusters of populations is given by


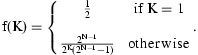


In other words, the prior probability of heterogeneity in allelic effects between populations is 0.5. Furthermore, when there is heterogeneity between populations, the number of clusters has a geometric distribution, such that *f*(*K*)/*f*(*K* + 1) = 2. This prior model gives greater probability to a partition with few clusters of populations. This is consistent with a prior belief that allelic effects are most likely to vary between broad ethnic groups, but are less likely to vary between more closely related populations.

Given *K*, each population is equally likely, *a priori*, to be a cluster centre, and the cluster allelic effects have a prior *N*(µ,σ) distribution, independent of **C**, where µ has a prior uniform distribution and σ has a prior exponential distribution with expectation 1. The weak joint prior density *f*(**ψ**,µ,σ) is readily overwhelmed by the data, and has been selected for computationally efficiency. Combining the components of the prior density function, it follows that


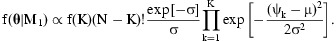


### MCMC ALGORITHM

It is not possible to evaluate the marginal likelihood *f*(**b**,**s**|*M*) directly. However, consider the joint posterior density of 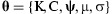 under the model *M*, given by


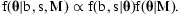
(4)

This density appears in the integrand of Equation (1) and can be approximated by means of a Metropolis–Hastings MCMC algorithm [Hastings, 1970; Metropolis et al., 1953]. The dimensionality of **θ** depends on the number of clusters of populations and can be addressed by incorporating a birth-death process for *K* by means of a reversible-jump step in the MCMC algorithm [Green, 1995]. In each iteration of the algorithm, candidate parameter values, **θ**^′^, are proposed by making “small” changes to the current set, as described in Supplementary Methods. The proposed parameter values are then accepted in place of **θ**′ with probability proportional to *f*(**θ**′|**b**,**s**,*M*)/*f*(**θ**|**b**,**s**,*M*); otherwise the current set is retained.

The MCMC algorithm is run for an initial burn-in period to allow convergence from randomly assigned starting values for **θ**. Convergence is assessed using standard diagnostics [Gammerman, 1997]. After convergence, each set of parameter values accepted or retained by the algorithm represents a draw from the posterior distribution *f*(**θ**|**b**,**s**,*M*). To reduce autocorrelation between consecutive draws of **θ**, the sampled set of parameter values is recorded at only every *t*th iteration of the algorithm, for some suitably large *t*.

Over *R* recorded outputs from the MCMC algorithm, with parameter values denoted 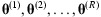, the marginal likelihood *f*(**b**,**s**|*M*) is approximated by


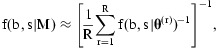


the harmonic mean of sampled likelihood values [Newton and Raftery, 1994]. In this expression,


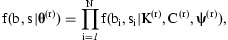


where 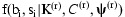 is given by Equation (3) for parameter values in 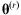. An estimate of the Bayes' factor, Λ, can then be obtained from two independent runs of the MCMC algorithm, once each under model *M*_0_ and *M*_1_.

The interpretation of the Bayes' factor depends on our prior beliefs about SNP association with the trait under investigation. On the basis of one million independent loci across the genome, plausible prior odds might be of the order of 10^4^−10^6^ against association [The Wellcome Trust Case Control Consortium, 2007]. Consequently, a Bayes' factor of the same order of magnitude would be necessary to provide convincing evidence of association [Stephens and Balding, 2009]. Alternatively, we could approximate the Bayesian false-discovery probability [Wakefield, 2007], and could vary the prior probability of association of each SNP according to annotation and/or minor allele frequency [Wang et al., 2005].

Output from the MCMC algorithm can be used directly to approximate the posterior distribution of the allelic effect, β_*i*_, in the *i*th population. Over *R* outputs, the posterior mean of this distribution is given by


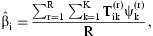


where 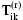 and 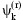 are parameter values in 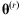.

Output from the algorithm can also be used to approximate the posterior probability of heterogeneity in allelic effects between populations under the alternative model of SNP association with the trait, given by the proportion of MCMC outputs for which *K* is greater than one. The prior model, *f*(*K*), assumes allelic effects to be equally likely to be homogeneous or heterogeneous across populations, so that *f*(*K* = 1) = *f*(*K*>1) = 0.5. Thus, a posterior probability of heterogeneity of greater than 0.95 would provide strong evidence of a deviation from homogeneity in allelic effects across populations. In this case, the posterior probability of heterogeneity in allelic effects between any given pair of populations can be approximated by the proportion of MCMC outputs for which they are assigned to different clusters of the Bayesian partition model. These probabilities can be used to construct a dendogram to represent the similarity between populations in terms of relatedness and allelic effects by application of average-linkage hierarchical clustering techniques [Hartigan, 1975].

### SOFTWARE AVAILABILITY

The MANTRA software has been developed to implement two independent runs of the MCMC algorithm, once each under *M*_0_ and *M*_1_. For each variant, and each population, MANTRA requires the following information: (i) the effect allele; (ii) the estimated effect allele frequency; (iii) the estimated allelic effect (log-odds ratio in the context of a dichotomous phenotype) and the corresponding standard error. For each variant, the software will estimate the Bayes' factor, Λ, in favor of association and summarize the output of the MCMC algorithm. MANTRA is available, as a suite of executables, on request from the author.

The run-time of the algorithm, per SNP, depends crucially on the number of studies, but is feasible on the scale of the whole genome through efficient parallel processing. For example, application of the MANTRA software to the meta-analysis of 28 transethnic GWAS, imputed up to 2.5 million SNPs from the International HapMap Project [The International HapMap Consortium, 2007], took less than 1 week with a cluster of 32 dedicated processors.

## RESULTS

In this section, I demonstrate the utility of MANTRA by application to association studies of T2D from five diverse ethnic groups [Waters et al., 2010]. I also present the results of a detailed simulation study to investigate the properties of MANTRA over a range of models of allelic effects between ethnic groups, primarily in terms of: (i) the power to detect association with a causal variant; and (ii) the localization of the causal variant within a 1-Mb region of the genome.

### EXAMPLE APPLICATION: TRANSETHNIC ASSOCIATION STUDIES OF T2D

**Fig. 1:**
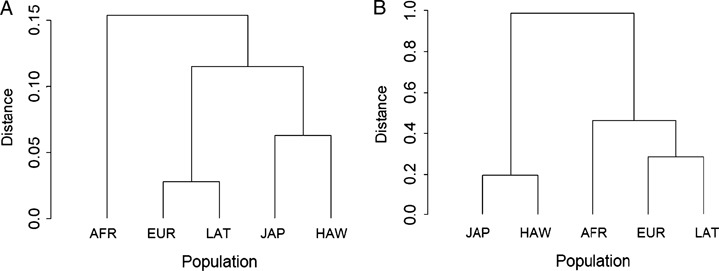
Dendograms to represent the relatedness between five populations from diverse ethnic groups. Population codes: African American (AFR); European American (EUR); Latinos (LAT); Japanese Americans (JAP); and Native Hawaiians (HAW). Panel **A** corresponds to the prior model of relatedness between populations, constructed on the basis of mean allele frequency differences across 19 variants. Panel **B** corresponds to the posterior similarity between populations in terms of relatedness and allelic effect at rs7754840, constructed from the posterior probabilities that each pair of populations appear in the same cluster of the Bayesian partition model.

There are more than 40 established loci associated with susceptibility to T2D, the majority of which have been identified through large-scale GWAS and meta-analysis in European-descent populations [Dupuis et al., 2010; Voight et al., 2010]. I have applied MANTRA to the results of five association studies of T2D [Waters et al., 2010], with samples ascertained from diverse populations: European Americans, African Americans, Latinos, Japanese Americans, and Native Hawaiians. A total of 6,142 cases and 7,403 controls were genotyped at 19 variants in established T2D loci (Table I). Relatedness between the populations was measured via the mean reference allele frequency difference over the 19 variants (Fig. 1A). Table I presents the results of two MANTRA analyses at each variant: (i) with an unconstrained number of clusters, *K*, of populations; and (ii) with a single cluster (*K =*1, i.e. fixed-effects). The results of the MANTRA analysis (*K* unconstrained) revealed overwhelming evidence of heterogeneity (99.2% posterior probability) in allelic effects between populations at just one locus: *CDKAL1*. The MANTRA analysis with *K* unconstrained at rs7754840 thus provided stronger evidence of association (log_10_ Bayes' factor = 11.0) than with fixed effects (*K =*1, log_10_ Bayes' factor = 8.9). The odds ratio of the risk allele at rs7754840 was noticeably stronger in the closely related Japanese American and Native Hawaiian populations than in European Americans, African Americans, and Latinos (Table II). It is not possible to formally partition within- and between-cluster heterogeneity in allelic effects at this variant. However, it was clear that the Japanese American and Native Hawaiians were often assigned to the same cluster of the Bayesian partition model at this variant (80.5% posterior probability), but rarely to a cluster containing the other populations (0.8% posterior probability), as demonstrated by the dendogram in Figure 1B.

**Table I:** Transethnic meta-analysis of five association studies of T2D at 19 variants in established susceptibility loci

					*K* unconstrained		
						
Locus	SNP	Chromosome	Position (bp)	Effect allele frequencies	log_10_ BF	*P*(heterogeneity)	*K* = 1 (fixed effect) log_10_ BF
*NOTCH2*	rs10923931	1	120,319,482	0.02–0.29	0.1	21.8%	0.0
*THADA*	rs7578597	2	43,586,327	0.75–0.99	0.8	25.4%	0.8
*PPARG*	rs1801282	3	12,368,125	0.89–0.97	0.8	55.2%	0.2
*ADAMTS9*	rs4607103	3	64,686,944	0.61–0.73	−0.3	9.2%	−0.3
*IGF2BP2*	rs4402960	3	186,994,381	0.27–0.49	3.3	24.6%	3.3
*WFS1*	rs10010131	4	6,343,816	0.59–0.98	2.0	70.1%	1.6
*CDKAL1*	rs7754840	6	20,769,229	0.29–0.55	11.0	99.2%	8.9
*JAZF1*	rs864745	7	28,147,081	0.51–0.77	7.4	22.7%	7.3
*SLC30A8*	rs13266634	8	118,253,964	0.60–0.89	3.7	11.0%	3.8
*CDKN2A/B*	rs2383208	9	22,122,076	0.56–0.85	5.0	15.6%	5.3
*HHEX*	rs1111875	10	12,368,016	0.28–0.74	0.4	32.2%	0.1
*TCF7L2*	rs7903146	10	94,452,862	0.04–0.28	17.0	21.9%	16.9
*CDC123*	rs12779790	10	114,748,339	0.14–0.18	1.3	16.1%	1.1
*KCNQ1*	rs2237895	11	2,813,770	0.20–0.42	1.7	13.3%	1.8
*KCNQ1*	rs2237897	11	2,815,122	0.62–0.95	3.9	13.7%	3.8
*KCNJ11*	rs5219	11	17,366,148	0.09–0.37	4.0	20.1%	3.8
*TSPAN8*	rs7961581	12	69,949,369	0.21–0.29	−0.3	13.2%	−0.4
*FTO*	rs8050136	16	52,373,776	0.20–0.43	−0.3	10.0%	−0.3
*HNF1B*	rs4430796	17	33,172,153	0.31–0.65	0.4	48.0%	0.1

Two MANTRA analyses are performed at each variant: (i) with an unconstrained number of clusters, *K*, of populations; and (ii) with a single cluster (*K =*1, i.e. fixed-effects). For each analysis, the log10 Bayes' factor (BF) in favor of association is presented. For the analysis with *K* unconstrained, the posterior probability of heterogeneity in allelic effects, *P*(heterogeneity), is also presented. T2D, type 2 diabetes.

**Table II:** Transethnic meta-analysis of five association studies of T2D at rs7754840 in the *CDKAL1* locus

Population	Sample size cases/controls	Effect allele frequency	Posterior median odds ratio (95% credibility interval)
European Americans	533/1,006	0.29	1.12 (1.03–1.38)
Latinos	2,220/2,184	0.31	1.10 (1.02–1.21)
African Americans	1,077/1,469	0.55	1.08 (0.95–1.19)
Japanese Americans	1,736/1,761	0.40	1.36 (1.23–1.48)
Native Hawaiians	576/983	0.52	1.36 (1.22–1.50)
Fixed-effects analysis	6,142/7,403		1.19 (1.13–1.25)

Two MANTRA analyses are performed: (i) with an unconstrained number of clusters, *K*, of populations; and (ii) with a single cluster (*K =*1, i.e. fixed-effects). Posterior median odds ratios and 95% credibility intervals for each ethnic group are obtained for *K* unconstrained. The fixed-effects posterior median odds ratio and 95% credibility interval is obtained for *K* = 1, assuming the same allelic effect across all five ethnic groups. T2D, type 2 diabetes.

### SIMULATION STUDY

**Fig. 2:**
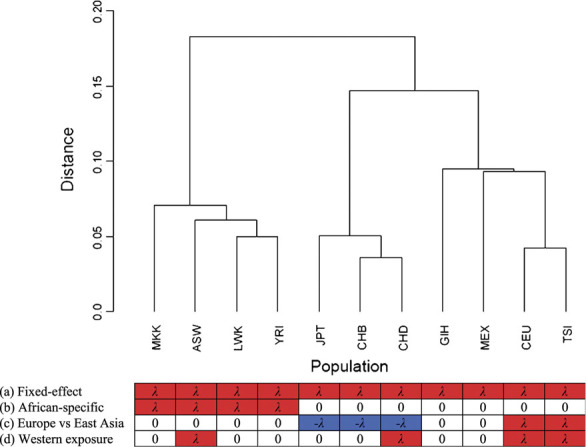
Dendogram to represent the relatedness between 11 diverse populations from Phase III of the International HapMap Project and the models of heterogeneity in allelic effects between them considered in the simulation study. Population codes: African ancestry in Southwest USA (ASW); Utah residents with North and Western European ancestry (CEU); Han Chinese in Beijing (CHB); Chinese in Metropolitan Denver (CHD); Gujarati Indians in Houston (GIH); Japanese in Tokyo (JPT); Luhya in Webuye, Kenya (LWK); Mexican ancestry in Los Angeles (MEX); Maasai in Kinyawa, Kenya (MKK); Toscani in Italy (TSI); and Yoruba in Ibadan, Nigeria (YRI). The relatedness between populations was measured by means of the mean allele frequency difference at 10,000 independent autosomal variants across the genome. The four models of heterogeneity are parameterised in terms of population-specific allelic effects, λ, and correspond to: (**a**) transethnic fixed-effect; (**b**) African-specific effect; (**c**) European and East Asian opposing effects; and (**d**) Western exposure effect.

Phase III of the International HapMap Project (HMP3) provides a reference panel of haplotypes at approximately 1.6 million variants, genomewide, obtained from 1,184 samples ascertained from 11 populations of European, Asian, and African descent [The International HapMap Consortium, 2010]. The relatedness between the populations, as measured via the mean allele frequency difference at 10,000 independent autosomal variants across the genome, is presented by means of the dendogram in Figure 2. In order to investigate the properties of MANTRA for detecting association with a quantitative trait, and fine-mapping the causal variant, I consider a range of models of heterogeneity in allelic effects between the populations, described in the four panels of Figure 2: (a) transethnic fixed-effect; (b) African-specific effect; (c) European and East-Asian opposing effects; and (d) Western exposure effect. In model (a), there is no heterogeneity in allelic effects at the causal variant between populations. In model (b), the causal variant has the same allelic effect in the four African descent populations (MKK, ASW, LWK, and YRI), but no effect in any of the other ethnic groups. In model (c), the causal variant has opposing allelic effects, of the same magnitude, in European-descent (CEU and TSI) and East-Asian descent populations (CHB, CHD, and JPT), but no effect in the other ethnic groups. Finally, in model (d), the causal variant has the same effect in those populations living in Europe or the USA (ASW, CHD, CEU, and TSI), but not in any other area. Such heterogeneity could occur, for example, when genotypes at the causal variant interact with exposure to a Western diet. In model (d), therefore, the most closely related populations do not share the most similar allelic effects, and thus offers the opportunity to test the sensitivity of MANTRA to this prior assumption.

For each model, I consider a range of population-specific allelic effect sizes, denoted λ_*P*_ (Fig. 2). For each allelic effect size, I then generate 1,000 replicates of data using the following approach:

1. Select a causal variant at random from HMP3, provided that it has a minor allele frequency of at least 1% in at least one population. Select one allele at this variant as the mean phenotype “increaser.” Consider all variants within 100 kb, up- and down-stream, as part of the analysis region.
2. For population *P*, simulate a cohort of 1,000 individuals by selecting pairs of reference haplotypes, at random, from HMP3. Record the genotypes of each individual at each variant within the analysis region. Simulate the phenotype of each individual from a unit variance Gaussian distribution, with mean given by λ_*P*_*g*_*i*_, where *g*_*i*_ is the number of increaser alleles, according to the model of heterogeneity. Repeat for each population.
3. For each population, estimate the effect of the increaser allele (assuming an additive model) and the corresponding standard error, *b*_*P*_ and *s*_*P*_, at each variant in the analysis region.
4. Perform three meta-analyses at each variant across populations using MANTRA: (i) assuming a single cluster of populations (*K* = 1, i.e. transethnic fixed-effect); (ii) assuming each population in assigned to a different cluster (*K* = *N*, i.e. random effects); and (iii) with the number of clusters of populations unconstrained. For each analysis, record the following summary statistics: (i) the Bayes' factor at the causal variant; (ii) the rank of the Bayes' factor at the causal variant among all variants in the analysis region; and (iii) the distance between the causal variant and the variant with the largest Bayes' factor in the analysis region (i.e. location error).

#### Performance of MANTRA under the null model of no association

Table III presents summary statistics for the two MANTRA analyses (*K* = 1 and *K* unconstrained) under the null model of no effect of the causal variant in any population (λ = 0). There are no discernable differences between the three analyses in terms of the evidence in favor of association at the causal variant, the location error, or the rank of the Bayes' factor at the causal variant. Given the size of the analysis region (100 kb up- and down-stream of the causal variant), the results are consistent with the expected location error of 50 kb. Furthermore, given that the density of variants in HMP3 is approximately one per 2 kb, the results are consistent with the expected median rank of the causal variant of 50.

**Table III:** Summary statistics for three MANTRA analyses (*K* = 1, *K* = *N*, and *K* unconstrained) under the null model of no effect of the causal variant in any population

Summary statistic	*K* = 1 (fixed effect)	*K* = *N* (random effect)	*K* unconstrained
Probability that BF>1 at the causal variant	0.09	0.12	0.09
Probability that BF>10 at the causal variant	0.00	0.01	0.01
Probability that BF>10^5^ at the causal variant	0.00	0.00	0.00
Mean location error of the causal variant (kb)	50.95	49.64	48.61
Median rank of BF at the causal variant	50.0	46.5	47.5
Probability that the causal variant has the largest BF	0.00	0.00	0.00

Evidence in favor of association is assessed by means of the BF. BF, Bayes' factor.

#### Power

**Fig. 3:**
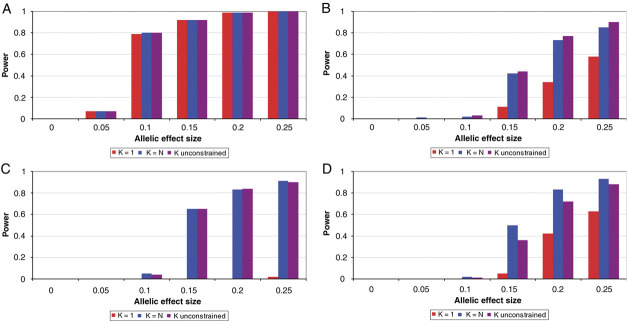
Power of three MANTRA analyses (*K* = 1, *K* = *N*, and *K* unconstrained), as a function of the allelic effect size, to detect evidence in favor of association at the causal variant at a Bayes' factor of 10^5^. Panels correspond to four models of heterogeneity in allelic effects between the populations: (**A**) transethnic fixed-effect; (**B**) African-specific effect; (**C**) European and East-Asian opposing effects; and (**D**) Western exposure effect.

Figure 3 presents the power of the three MANTRA analyses (*K* = 1, *K* = *N*, and *K* unconstrained), as a function of the allelic effect size, to detect evidence in favor of association at the causal variant at a Bayes' factor of 10^5^. This threshold corresponds to prior odds of 10^5^ against association of any variant with the phenotype [The Wellcome Trust Case Control Consortium, 2007], but has no impact on the relative performance of the three analyses. Figure 3A presents the power of the three analyses under the transethnic fixed-effect model where the allelic effect of the causal variant is the same in all populations. Consequently, there is no discernable difference in power between the three MANTRA analyses. In the remaining panels of Figure 3, corresponding to models of heterogeneity in allelic effects between populations, the fixed-effect MANTRA analysis (*K* = 1) has substantially less power than the random-effect analysis (*K* = *N*) or the unconstrained analysis. The difference is particularly striking for the model of European and East Asian opposing effects (Fig. 3C). In this scenario, the allelic effects in these two ethnic groups effectively cancel each other out, with the result that the fixed-effects MANTRA analysis has minimal power to detect association. Figure 3D, corresponding to the Western exposure effect model, demonstrates the increased power of the unconstrained MANTRA analysis, even when allelic effect heterogeneity does not adhere to our prior assumption of relatedness between populations. Figure 3B, corresponding to the African-specific effect model highlights reduced power for the random-effect MANTRA analysis (*K* = *N*) compared to the unconstrained analysis. There is no discernable difference in power between these two analyses for the model of European and East Asian opposing effects (Fig. 3C), where we would expect four clusters of populations to best explain the heterogeneity in allelic effects. However, in Figure 3D, corresponding to the Western exposure effect model, the random-effect MANTRA analysis is most powerful. These results suggest that the unconstrained MANTRA analysis has greatest gains in power over the random-effect analysis when the pattern of heterogeneity in allelic effects between populations is well represented by the prior Bayesian partition model, and when there are fewer clusters.

#### Heterogeneity in allelic effects between populations

**Fig. 4:**
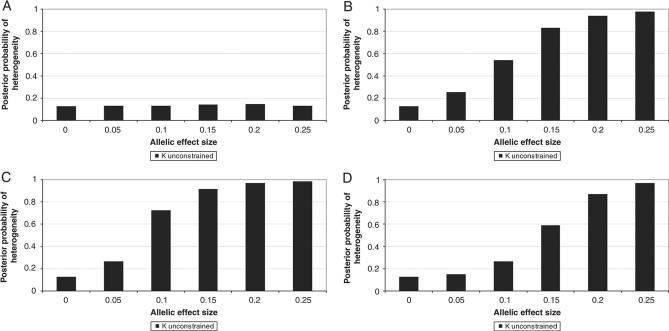
Mean posterior probability of heterogeneity from the MANTRA analysis with *K* unconstrained, as a function of the allelic effect size. Panels correspond to four models of heterogeneity in allelic effects between the populations: (**A**) transethnic fixed-effect; (**B**) African-specific effect; (**C**) European and East-Asian opposing effects; and (**D**) Western exposure effect.

Figure 4 presents the mean posterior probability of heterogeneity from the MANTRA analysis with *K* unconstrained, as a function of the allelic effect size. Figure 4A presents the mean posterior probability under the transethnic fixed effect model, and thus shows no evidence of heterogeneity, relative to the prior probability of 0.5, irrespective of allelic effect size. However, in each of the models of heterogeneity between populations presented in the remaining panels of Figure 4, the mean posterior probability increases with allelic effect size, as expected.

#### Localization

**Fig. 5:**
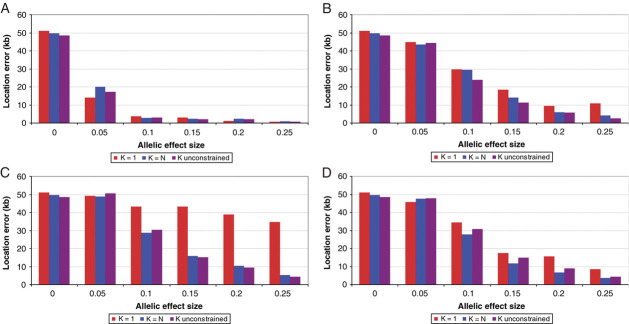
Mean location error (kb) of three MANTRA analyses (*K* = 1, *K* = *N*, and *K* unconstrained), as a function of the allelic effect size. Panels correspond to four models of heterogeneity in allelic effects between the populations: (**A**) transethnic fixed-effect; (**B**) African-specific effect; (**C**) European and East-Asian opposing effects; and (**D**) Western exposure effect.

**Fig. 6:**
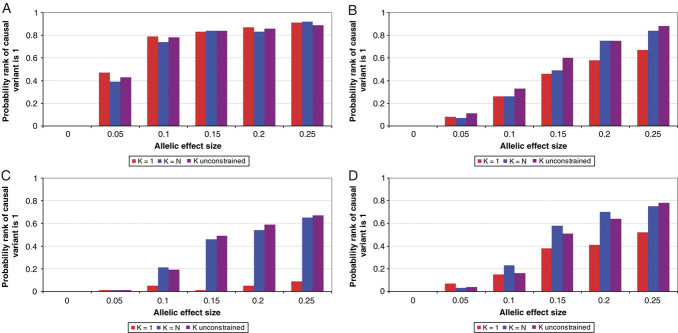
Probability that the causal variant has the largest Bayes' factor in favor of association from three MANTRA analyses (*K* = 1, *K* = *N*, and *K* unconstrained), as a function of the allelic effect size. Panels correspond to four models of heterogeneity in allelic effects between the populations: (**A**) transethnic fixed-effect; (**B**) African-specific effect; (**C**) European and East-Asian opposing effects; and (**D**) Western exposure effect.

Figure 5 presents the mean location error (kb) of the three MANTRA analyses (*K* = 1, *K* = *N*, and *K* unconstrained) as a function of the allelic effect size. As expected, there is no discernable difference in location error between the three analyses under the transethnic fixed effect model (Fig. 5A). In the remaining panels of Figure 5, corresponding to models of heterogeneity in allelic effects between populations, the fixed-effect MANTRA analysis (*K* = 1) has substantially less precision for fine-mapping than the random-effect MANTRA analysis (*K* = *N*) or the unconstrained analysis. The same conclusions are reached by considering the probability that the causal variant has the largest Bayes' factor in favor of association in the 200-kb analysis region (Fig. 6). In the same way as for power, the most striking differences in localization between the three MANTRA analyses were observed for the most extreme model of heterogeneity between populations, namely European and East Asian opposing allelic effects (Figs. 5C and 6C). Furthermore, the unconstrained MANTRA analysis demonstrated greater precision for fine-mapping than the random-effect analysis (*K* = *N*), unless the pattern of heterogeneity in allelic effects between populations is poorly represented by the prior Bayesian partition model.

#### Impact of the sample size of an outlying cluster

**Fig. 7:**
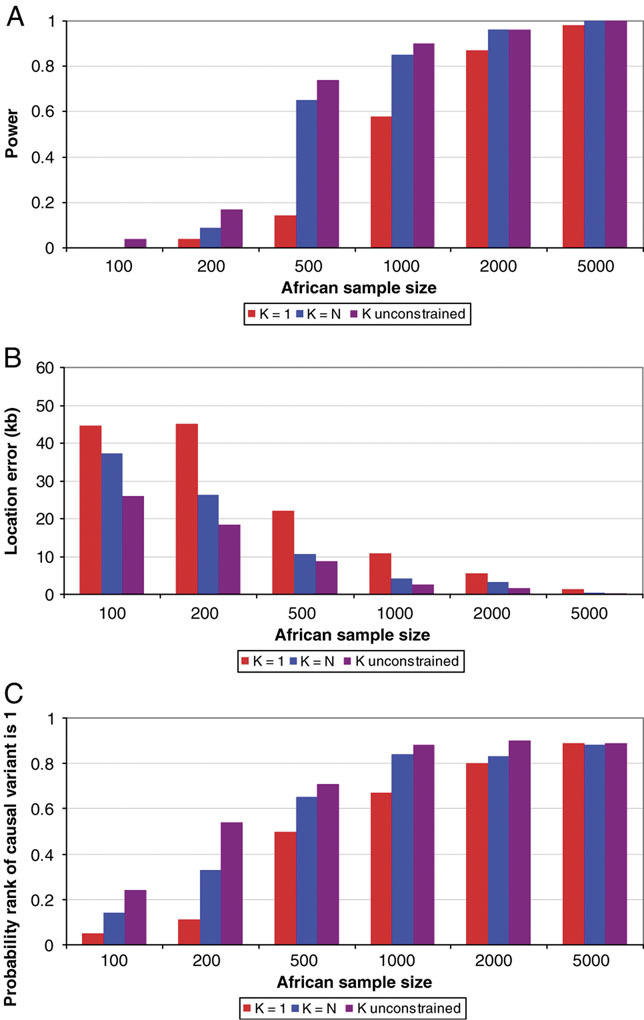
Summary of three MANTRA analyses (*K* = 1, *K* = *N*, and *K* unconstrained), as a function of the sample size of studies from populations of African descent (MKK, ASW, LWK and YRI). These simulations assume an African-specific effect of λ = 0.25. The three panels correspond to: (**A**) power to detect evidence in favor of association at the causal variant at a Bayes' factor of 10^5^; (**B**) mean location error (kb); and (**C**) probability that the causal variant has the largest Bayes' factor in favor of association.

In order to assess the impact of sample size of studies in an outlying cluster of the Bayesian partition model, I have repeated simulations of an African-specific effect (with λ = 0.25 in all African populations). Figure 7 presents summary statistics for the three MANTRA analyses (*K* = 1, *K* = *N*, and *K* unconstrained) as a function of the sample size of the four studies of African descent (MKK, ASW, LWK, and YRI). The three panels demonstrate that the advantage of the unconstrained MANTRA analysis over fixed-effect (*K* = 1) analysis and random-effect (*K* = *N*) analysis is unaffected by sample size, both in terms of power (Fig. 7A) and of precision of fine-mapping (Fig. 7B and C).

#### Assessment of the impact of transethnic data on power and localization

**Fig. 8:**
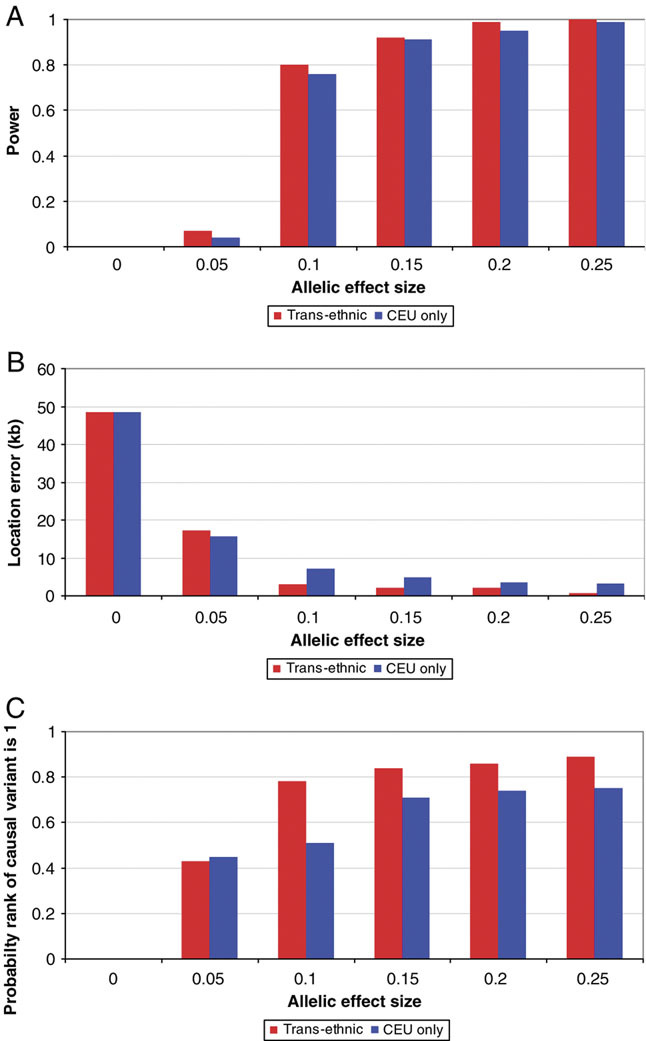
Summary of MANTRA analysis with *K* unconstrained, as a function of the allelic effect size, for 11 GWAS from the same CEU population compared with 11 GWAS from different transethnic populations. These simulations incorporate no heterogeneity in allelic effects between populations, i.e. a transethnic fixed-effect model. The three panels correspond to: (**A**) power to detect evidence in favor of association at the causal variant at a Bayes' factor of 10^5^; (**B**) mean location error (kb); and (**C**) probability that the causal variant has the largest Bayes' factor in favor of association.

In order to assess the benefits of transethnic GWAS for the detection and fine-mapping of novel loci for complex traits, I have repeated simulations of the transethnic fixed-effect model under two scenarios: (i) 11 GWAS of 1,000 individuals, each ascertained from a different HMP3 population; and (ii) 11 GWAS of 1,000 individuals, each ascertained from the same CEU population. Figure 8 presents summary statistics for the MANTRA analysis (*K* unconstrained), as a function of the allelic effect, in each of the two scenarios. Figure 8A highlights no discernable difference in power between the two scenarios, which would be expected given that the causal variant has the same allelic effect in all populations. Within any single replicate of data, differences in the Bayes' factor in favor of association reflect variation in allele frequencies at the causal variant across populations. The remaining panels of Figure 8 demonstrate that the transethnic GWAS strategy has improved precision for fine-mapping, despite the transethnic fixed-effect, with lower mean location error and higher probability that the causal variant has the largest Bayes' factor in favor of association in the 200-kb analysis region. These improvements in mapping resolution, without a corresponding increase in power to detect association, reflect differences in LD patterns with the causal variants between ethnic groups, which cannot be leveraged from using GWAS ascertained from the same population.

## DISCUSSION

Meta-analysis of GWAS of primarily European-descent populations has been an extremely efficient approach to identifying novel loci contributing effects to complex traits by increasing sample size without de novo genotyping. The underlying assumption of traditional fixed-effects meta-analysis is that the allelic effect of a given variant is homogeneous across studies. For GWAS ascertained from the same or closely related populations, such an assumption is reasonable. The recent shared ancestry of these populations increases the likelihood that they will have the same underlying common causal variants, similar allele frequency spectra and local LD profiles. Exposure to potential nongenetic risk factors, such as diet, smoking, and pollution, which may interact with genotypes at causal variants, is also likely to be similar in European populations, further reducing the prospect of heterogeneity in allelic effects between them.

With the increasing availability of GWAS from more diverse populations, transethnic meta-analysis might be expected to further increase power to detect additional complex trait loci with ever more modest effects. However, with more diverse populations, less recent shared ancestry introduces greater opportunity for genetic heterogeneity, both in terms of the underlying causal variants and their allelic effect on the trait. Standard statistical methodology exists for assessing the evidence of heterogeneity in fixed-effects meta-analysis, such as *I*^2^ and Cochran's *Q*-Statistic [Higgins and Thompson, 2002; Huedo-Medina et al., 2006; Ioannidis et al., 2007], and can thus be used to highlight populations with outlying allelic effects. In the presence of such allelic heterogeneity, these outlying populations could be removed, although potentially resulting in a reduction in power. On the other hand, random-effects meta-analysis, which assumes that each population has a different underlying allelic effect, can be used to overcome the problem of heterogeneity. However, this is also unsatisfactory since we expect populations from the same ethnic group to be more homogeneous than those that are more distantly related. A plausible alternative approach to transethnic meta-analysis would be to make use of a hierarchical model in which the allelic effect estimates for each population are considered as a function of indicator variables that represent ethnic group. This approach has the advantage over random-effects meta-analysis of allowing for similarity in allelic effects across populations from the same ethnic group. However, the assignment of populations to ethnic groups is prespecified by this prior classification, and cannot borrow from the observed allelic effect estimates to inform clustering.

In this article, I have addressed the challenges of allelic effect heterogeneity posed by transethnic meta-analysis of GWAS by considering the relatedness between the populations from which they have been ascertained. The Bayesian partition model provides a natural framework to take advantage of the expectation that more closely related populations are more likely to have similar allelic effects than those from diverse ethnic groups. The key advantage of this approach over a purely random effects analysis is that we can model the allelic heterogeneity between ethnic groups. Specifically, populations are clustered according to their “prior” similarity in terms of relatedness, typically using genomewide data to approximate their shared ancestry, and their semblance in terms of allelic effects at a specific variant under investigation. Populations within the same cluster are assumed to have the same underlying allelic effects at this variant. However, different clusters need not have the same underlying allelic effect. MANTRA can thus be thought of as a *hybrid* meta-analysis, incorporating both fixed (i.e. *within* cluster) and random (i.e. *between* clusters) effects.

The application of MANTRA to transethnic association studies of T2D at 19 variants in established susceptibility loci highlighted little evidence of heterogeneity in allelic effects between five diverse populations. However, there was overwhelming evidence of heterogeneity at rs7754840 in the *CDKAL1* locus. Allelic effects on T2D were in the same direction in all populations, but were considerably stronger in the closely related Japanese Americans and Native Hawaiians than in European Americans, Latinos, or African Americans. Such heterogeneity could arise as a result of multiple causal variants in *CDKAL1*, one of which is specific to the Japanese American and Native Hawaiian populations. However, this pattern of allelic effects could also arise with a single causal variant as a result of differences in the local LD structure between populations. In particular, rs7754840 may better capture the causal variant in the Japanese American and Native Hawaiian populations, which is not implausible given their recent shared ancestry. Interestingly, the lack of heterogeneity in allelic effects at the majority of established T2D loci suggests that the underlying causal variants are the same across ethnic groups, and hence pre-date any “out of Africa” population migration, which cannot be well modeled by “synthetic association” of multiple rare alleles [Dickson et al., 2010].

The results of the simulation study highlight that the hybrid meta-analysis implemented in MANTRA outperforms fixed-effects, both in terms of power to detect association, and localization of causal variants, over a range of models of heterogeneity in allelic effects between diverse populations. The greatest gains in power are achieved under a model of heterogeneity in which the causal variant has opposing effects in different populations, although it is not clear how realistic this scenario is likely to be. Under a model of homogeneous allelic effects across ethnic groups, there is no discernible loss in power or fine-mapping accuracy for the hybrid MANTRA analysis over fixed-effects meta-analysis. Furthermore, there are noticeable improvements in the localization of causal variants with MANTRA when applied to meta-analysis of transethnic, rather than intraethnic GWAS, even under a model of homogeneous allelic effects across populations. These improvements in the resolution of fine-mapping reflect transethnic differences in local LD patterns which cannot be leveraged from GWAS ascertained from the same population. The results of the simulation study also highlight advantages of the hybrid MANTRA analysis over random-effects meta-analysis, both in terms of power and localization of causal variants, when heterogeneity in allelic effects is well represented by the prior Bayesian partition model. Output from the MANTRA MCMC algorithm can also be used to represent the pattern of heterogeneity in allelic effects between populations, which cannot be achieved with random-effects meta-analysis.

The use of diverse populations from multiple ethnic groups will play an essential role in future GWAS. European-descent populations contain only a subset of human genetic variation, and thus cannot be used to identify causal variants across ethnic groups. This is particularly relevant for lower frequency causal variants, which are more likely to be population specific, but which have been hypothesized to contribute substantially to the missing heritability of complex traits [Frazer et al., 2009]. The reduced bias of GWAS genotyping products toward European genetic variation, and the increasing availability of large-scale resequencing reference panels from a wide range of ethnic groups, greatly improves the prospects of imputation across diverse populations. Efficient and powerful statistical methodology for the analysis of transethnic GWAS, such as the MANTRA software developed here, thus shows great promise for future improvements in our understanding of the genetic architecture of complex human traits.
