# Supplementary material for: Transethnic Meta-Analysis of Genomewide Association Studies
Source: Genet Epidemiol. 2011 Nov 28;35(8):809–22. doi: 10.1002/gepi.20630 (PMC3460225; doi:10.1002/gepi.20630)
Supplement: Supplementary file 1 [file gepi0035-0809-SD1.doc]

**Trans-ethnic meta-analysis of genome-wide association studies**

**Andrew P. Morris**

Wellcome Trust Centre for Human Genetics, University of Oxford, Oxford, United Kingdom

**Supplementary Material**

**Supplementary Methods**

**Details of the MCMC algorithm**

A reversible-jump Metropolis-Hastings MCMC algorithm has been developed to approximate the posterior density function *f*(**θ**|**b**,**s**,*M*), given by

$$f\left(\mathbf{\theta}\left|\mathbf{b},\mathbf{s},M\right.\right)\propto f\left(\mathbf{b},\mathbf{s}\left|\mathbf{\theta}\right.\right)f\left(\mathbf{\theta}\left|M\right.\right)$$

for model *M*, where **θ** = {*K*,**C**,**ψ**,*μ*,*σ*}, as defined in **Methods**. For each iteration of the algorithm, a new set of parameter values, **θ**′, is proposed according to predetermined weights, **w**, chosen to optimize mixing and convergence (**Supplementary Table 1**). The proposed parameter values are substituted for the current set, provided that

,

where *ε* is a standard uniform random variable and *δ* denotes the Hastings ratio of proposal probabilities to be defined below. Otherwise, the current set of parameters is retained. The possible changes to the parameter set are summarized below, where *ε* is a standard uniform random variable.

**Change 1: propose a cluster birth**

The proposed number of clusters is given by *K* = *K*+1. Select a position, *k**, at random for the new cluster in the list of ordered cluster centres. Select a population *P_i_*, that is not already a cluster centre, *C_k_** = *P_i_*. Generate a new allelic effect, *ψ*′*_k_**, from a N(*μ*,*σ*) distribution. Then the remaining cluster centres and cluster allelic effects are given by,

*C*′*_k_* = *C_k_* and *ψ*′*_k_* = *ψ_k_* if *k* < *k**,

and

*C*′*_k+1_* = *C_k_* and *ψ*′*_k+1_* = *ψ_k_* if *k* > *k**.

To ensure reversibility,

.

**Change 2: propose a cluster death**

The proposed number of clusters is given by *K* = *K*-1. Select a cluster, *k**, at random for death. The proposed cluster centres and cluster allelic effects are then given by,

*C*′*_k_* = *C_k_* and *ψ*′*_k_* = *ψ_k_* if *k* < *k**,

and

*C*′*_k_* = *C_k+1_* and *ψ*′*_k_* = *ψ_k+1_* if *k* > *k**.

To ensure reversibility,

.

**Change 3: propose a cluster centre swap**

Select a pair of clusters, *k*_1_ and *k*_2_, at random. The proposed cluster centres and cluster allelic effects are then given by

*C*′*_k1_* = *C_k2_* and *ψ*′*_k1_* = *ψ_k2_*,

and

*C*′*_k2_* = *C_k1_* and *ψ*′*_k2_* = *ψ_k1_*.

To ensure reversibility, *δ* = 1.

**Change 4: propose a cluster centre change**

Select a cluster, *k*, at random. Select a population, *P_i_*, at random from those that are not already a cluster centre, so that *C*′*_k_* = *P_i_*. To ensure reversibility, *δ* = 1.

**Change 5: propose new cluster allelic effects**

For each cluster, *k*, in turn, propose a cluster allelic effect, *ψ*′*_k_* = *ψ_k_*+*ν_ψ_*(*ε*-0.5), where *ν_ψ_* denotes the maximum change in the parameter value. To ensure reversibility, *δ* = 1.

**Change 6: propose prior model hyperparameters**

First, propose a mean for the prior density of cluster allelic effects, *μ*′ = *μ*+*ν_μ_*(*ε*-0.5), where *ν_μ_* denotes the maximum change in the parameter value. To ensure reversibility, *δ* = 1. Next, propose a standard deviation for the prior density of cluster allelic effects, *σ*′ = *σ*+*ν_σ_*(*ε*-0.5), where *ν_σ_* denotes the maximum change in the parameter value. To ensure reversibility, *δ* = 1, and *σ*′ = -*σ* if *σ*′ < 0.

**Supplementary Table 1. Summary of possible changes to the current parameter set in the reversible-jump Metropolis-Hastings MCMC algorithm.** Changes are selected at random, according to weights, **w**.

| Change (*j*) | Proposal | Parameters | Weights *w_j_*(*K*) | | |
| --- | --- | --- | --- | --- | --- |
|  |  |  | *K* = 1 | 1 < *K* < *N* | *K* = *N* |
| 1 | Cluster birth | *K*, **C**, **ψ** | 0.42 | 0.25 | 0 |
| 2 | Cluster death | *K*, **C**, **ψ** | 0 | 0.25 | 0.56 |
| 3 | Cluster centre swap | **C**, **ψ** | 0 | 0.15 | 0 |
| 4 | Cluster centre | **C** | 0.25 | 0.15 | 0 |
| 5 | Cluster allelic effect | **ψ** | 0.25 | 0.15 | 0.33 |
| 6 | Hyper-parameters | *μ*, *σ* | 0.08 | 0.05 | 0.11 |
